# Age-Related Differences in Lipopolysaccharide-Induced Delirium-like Behavior Implicate the Distinct Microglial Composition in the Hippocampus

**DOI:** 10.3390/ijms26052055

**Published:** 2025-02-26

**Authors:** Congli Sun, Xiaomin Kang, Xirui Jia, Yuwei Wang, Lijia Zhao, Xinyu Sun, Anaerguli Abula, Lijie Liu

**Affiliations:** 1 Department of Physiology, School of Medicine, Southeast University, Nanjing 210009, China; 230208416@seu.edu.cn; 2 School of Life Science and Technology, Southeast University, Nanjing 210009, China; 220213913@seu.edu.cn (X.K.); 220223581@seu.edu.cn (X.J.); 3 School of Medicine, Southeast University, Nanjing 210009, China; 213211126@seu.edu.cn (Y.W.); 213210002@seu.edu.cn (L.Z.); 213210403@seu.edu.cn (X.S.); 213213732@seu.edu.cn (A.A.); 4 Jiangsu Provincial Key Laboratory of Critical Care Medicine, Department of Physiology, School of Medicine, Southeast University, Nanjing 210009, China

**Keywords:** age, delirium, microglial response, senescence

## Abstract

As the global population ages, the mechanisms underlying age-related susceptibility to delirium have attracted attention. Given the central role of microglia in the pathogenesis of inflammation-related delirium, we investigated the temporal dynamics of neurobehavioral changes and microglial responses, following lipopolysaccharide (LPS, 200 μg/kg) administration in young and old male C57BL/6 mice. Although a similar illness trajectory across 48 h post-treatment (HPT) was observed in both age groups, old-LPS mice exhibited worsened delirium-like behavior. At 48 HPT, in old but not young mice, significantly decreased hippocampal neuronal activity coincided with microglial overactivation. Widespread hippocampal microglial activation was present at 3 HPT but subsided by 12 HPT in young but not old mice, indicating a generally retarded but prolonged microglial response to LPS challenge in old mice. However, for both age groups, at 3 HPT, p16^INK4a^-negative microglia (with low abundance in the aged brain) exhibited comparable morphological activation, which was not observed for p16^INK4a^-positive microglia (highly abundant in the aged brain). These results suggest that age-related susceptibility to LPS-induced delirium-like behavior accompanied by different patterns of microglial response might implicate microglial composition shifts and that optimizing microglial composition represents a promising approach to reduce vulnerability to inflammatory challenge.

## 1. Introduction

Delirium is a profound neuropsychiatric syndrome characterized by an acute onset and a fluctuating course of inattention, impaired consciousness, and disturbance of cognition [1,2,3]. Accumulating data from clinical studies show that delirium is strongly associated with increased morbidity and long-term cognitive impairments affecting survivors’ quality of life; thus, this medical issue warrants special attention [3,4]. Although delirium can occur across the lifespan, advanced age has been well established as a significant predisposing factor [2,3,5]. With the dramatic increase in the aging population worldwide and the growing number of survivors of critical illness benefiting from rapid advances in medicine, the underlying mechanisms of age-related susceptibility to delirium have attracted increasing attention.

Microglia are the principal lifelong patrolling immune cells in the brain and serve as an indispensable part of the first line of defense against pernicious stimuli [6,7]. Microglial morphology is inextricably associated with microglial functional status [8,9,10,11]. Homeostatic microglia, which can be identified in the healthy brain by their highly ramified morphology characterized by long, thin processes and small soma, continuously monitor their own parcel of parenchyma to maintain brain homeostasis and ultimately brain function. Upon stimulation, homeostatic microglia undergo a dramatic morphological transformation from a highly ramified to an activated amoeboid-like phenotype characterized by an increased soma area, less ramified but thickened processes, and upregulation of CD68, a marker of activated microglia with phagocytic activity. With normal aging, similar to immune cells in the periphery, microglia undergo functional changes with age. Aged microglia not only express senescence protein markers, including p16^INK4a^ [12], but also exhibit dystrophic morphology characterized by the loss of long processes that are vital for communication with their environment accompanied by beaded or fragmented processes [13,14]. Prompt appropriate microglial activation may confer neuroprotection by clearing pathogens and cell debris, whereas sustained inappropriate microglial overactivation may contribute to multiple pathologies of neurodegenerative diseases [15,16,17]. Although there is substantial evidence indicating that microglia are key players in delirium pathogenesis [2,3,18,19], and that the maladaptive response of senescent microglia to stimuli is a potent contributor to increased vulnerability to age-related neurodegenerative diseases as well as worsened outcomes after injury [20,21], how microglial senescence is associated with increased vulnerability to acute delirium-like events arising during aging per se has rarely been addressed.

Systemic administration of the endotoxin lipopolysaccharide (LPS), the critical component of the outer membrane of Gram-negative bacteria, is a well-established and widely used paradigm in rodent research for investigating the mechanisms of delirium [22,23,24,25]. The hippocampus, a critical brain region for cognition, memory, and executive function [26,27,28], is particularly vulnerable to the potentially detrimental effects of inflammatory stimuli [29] and appears to play a critical role in the pathogenesis of delirium [30]. In this study, we administered LPS (200 μg/kg, intraperitoneally [i.p.]) to young (2-month-old) and old (10-month-old) male C57BL/6 mice and characterized the dynamics of the behavioral alterations, indicating impairments in attention, consciousness, and cognition, and the status of the hippocampal microglia in both age groups. We demonstrated that the greater susceptibility to LPS-induced delirium-like behavior in older mice was concomitant with aberrant time-course profiles of the microglial response, which may be attributed at least in part to microglial senescence, a phenomenon that contributes to shifts in microglial composition. Furthermore, aberrant neuronal activation accompanied by prolonged microglial overactivation might underlie the increased vulnerability to future neurocognitive deficits. Our observations suggest that strategies designed to minimize the proportion of senescent microglia (namely, remodeling the microglial composition) could be promising therapeutic options for reducing the incidence of delirium and delirium-related neurocognitive sequelae.

## 2. Results

### 2.1. Age-Related Differences in Lipopolysaccharide-Induced Delirium-like Behavior

**Figure 1:**
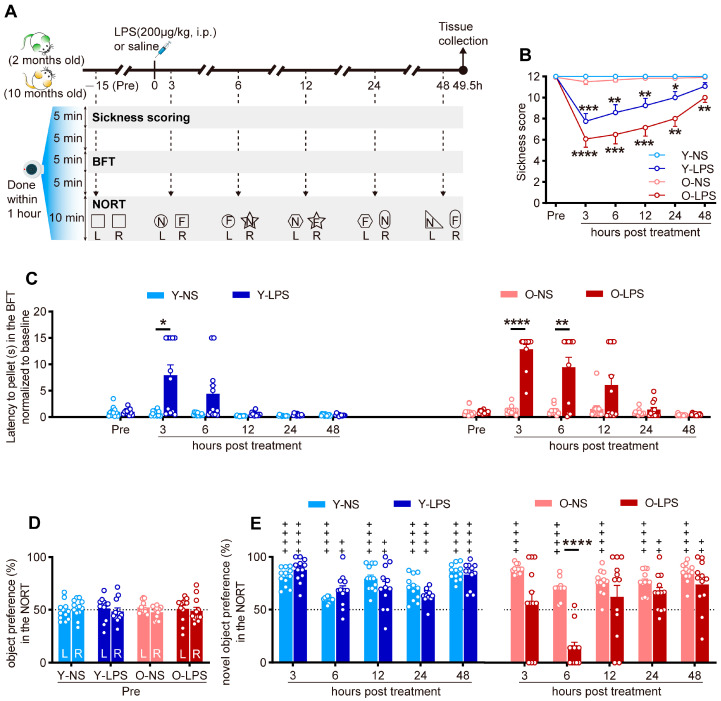
Age-related differences in behavioral changes following LPS treatment. (**A**) Schematic diagram of the treatment schedule and timeline for behavioral testing and tissue collection. LPS, lipopolysaccharide; Pre, pre-treatment; BFT, buried food test; NORT, novel object recognition test; N, novel object; F, familiar object; L, left side; R, right side. (**B**) Behavioral sickness response scores pre-treatment (Pre) and at 3, 6, 12, 24, and 48 h post-treatment in young and old mice. (**C**) Latency to pellet in the BFT at Pre, 3, 6, 12, 24, and 48 h post-treatment in young and old mice. The data were normalized to baseline (dividing each value by the average of the data obtained pre-treatment). (**D**) The percentage of preference for two identical objects (left and right) during the first session of the NORT performed pre-treatment. The dashed line represents the 50% chance level. (**E**) The percentage of preference for novel objects in the following sessions of the NORT performed at 3, 6, 12, 24, and 48 h post-treatment. The data in (**B**) were analyzed using two-way repeated-measures ANOVA followed by Tukey’s post hoc test (* *p* < 0.05; ** *p* < 0.01; *** *p* < 0.001; **** *p* < 0.0001, vs. the age-matched NS group). The data in (**C**,**E**) were analyzed using two-way repeated-measures ANOVA followed by Sidak’s post hoc test (* *p* < 0.05; ** *p* < 0.01; **** *p* < 0.0001, vs. age-matched NS group). The results of the ANOVA tests were listed in Supplementary Table S1. The data in (**D**) were analyzed using Student’s two-tailed *t* test for each group. The significance versus chance level (50%, denoted by the dashed line) in (**E**) was analyzed by one sample *t* test (^++^ *p* < 0.01; ^++++^ *p* < 0.0001). All of the data are expressed as the mean ± standard error of the mean (SEM) (*n* = 12 mice per group).

As illustrated in Figure 1A, time-course behavioral assessments, including sickness scoring, the buried food test (BFT), and the novel object recognition test (NORT), were performed prior to (Pre) and during the 48 h post-treatment (HPT) with normal saline (NS) or lipopolysaccharide (LPS). Both young and old LPS-treated mice presented similar illness trajectories, as indicated by significantly decreased sickness scores obtained at the designated test time points post-injection (Figure 1B). The latency to pellet in the BFT, a widely used index of attention and organized thinking [31,32,33], was significantly increased in young LPS-treated mice only at 3 HPT but was significantly increased in old LPS-treated mice at both 3 and 6 HPT (Figure 1C). The animals in all of the groups spent comparable amounts of time exploring each of the two identical objects located on opposite sides of the cage during the first session of NORT performed prior to injection (Figure 1D), excluding the potential side preference [34]. Interestingly, although all of the NS-treated and young LPS-treated mice interacted with the novel object significantly more than chance (50%) in each of the following sessions (i.e., showed a significant preference for the novel object), the old LPS-treated mice demonstrated no preference for the novel object at 6 HPT (Figure 1E), indicating that the old LPS-treated mice developed transient cognitive impairment [30,35]. The combination of the above observations not only confirmed that LPS injection could induce delirium-like behavioral changes in mice but also revealed that identical LPS treatment induced more severe episodes of delirium-like behavioral changes in old than in young mice.

### 2.2. Aberrant Neuronal Activity Associated with Prolonged Microglial Activation in the Hippocampus of Old Mice

As studies have shown that delirium is associated with changes in neuronal activity patterns of the hippocampus [36,37] and that the severity and duration of aberrant neuronal activation are closely related to the outcome of the condition [38], brain samples from 48 HPT mice were subjected to immunochemical staining for c-Fos (a high-resolution marker for neural activity), NeuN (a neuronal marker), and Iba1.

**Figure 2:**
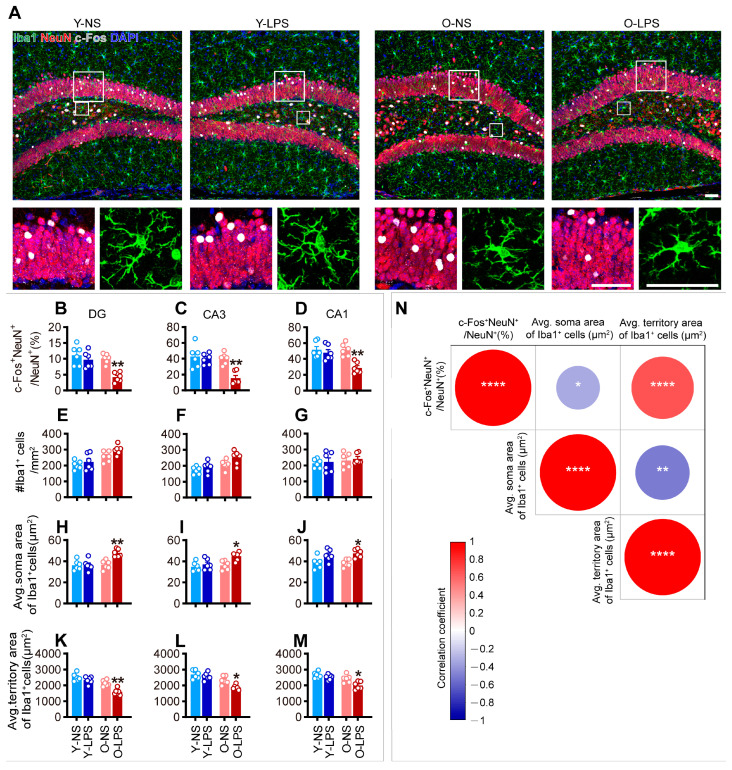
Aberrant neuronal activity and prolonged microglial alterations at 48 HPT in old mice. (**A**) Representative maximum intensity projections of confocal images of c-Fos-, NeuN-, and Iba1-labeled hippocampal DG subregions surveyed in all groups at 48 HPT. Higher magnification images of the solid line boxed areas are shown below the corresponding image. Scale bars = 50 μm. LPS, lipopolysaccharide. (**B**–**M**) Quantification of the percentage of c-Fos^+^ neurons (**B**–**D**), microglial density (**E**–**G**), average microglial soma area (**H**–**J**), and average microglial territory area (**K**–**M**) in the DG, CA3, and CA1. The data were analyzed using two-way ANOVA (detailed results were listed in Supplementary Table S2) with Tukey’s post hoc test (* *p* < 0.05; ** *p* < 0.01, vs. age-matched NS group). All of the data are expressed as the mean ± standard error of the mean (SEM) (*n* = 6 mice per group). (**N**) Correlation heatmap color-coded by the strengths of Pearson correlation coefficients between the percentage of c-Fos^+^ neurons, microglial soma area, and microglial territory area. Red circles indicate positive associations while blue circles indicate negative associations. Significance levels are given as * *p* ≤ 0.05; ** *p* ≤ 0.01; **** *p* ≤ 0.0001.

Figure 2A shows representative confocal images of the hippocampal DG subregions in each group. Compared with those in age-matched controls, the percentage of c-Fos^+^ neurons was comparable in young mice but was significantly lower in old mice (Figure 2B–D), indicating aberrant neuronal activation in the hippocampus of old LPS-treated mice at 48 HPT. Consistent with these observations, although no alterations in hippocampal microglial density were observed in mice of either age group (Figure 2E–G), increases in the soma area (Figure 2H–J) and decreases in the territory area (Figure 2K–M) were observed in old mice, suggesting prolonged activation of hippocampal microglia in old LPS-treated mice.

Linear regression analysis revealed a significant negative correlation between the percentage of c-Fos^+^ neurons and the microglial soma area, as well as a significant positive correlation between the percentage of c-Fos^+^ neurons and the microglial territory area in old mice (Figure 2N), indicating a potential relationship between aberrant neuronal activation and a perturbed microglial phenotype in the hippocampus of old LPS-treated mice.

### 2.3. Age-Related Differences in the Time-Course Profiles of the Hippocampal Microglial Response

**Figure 3:**
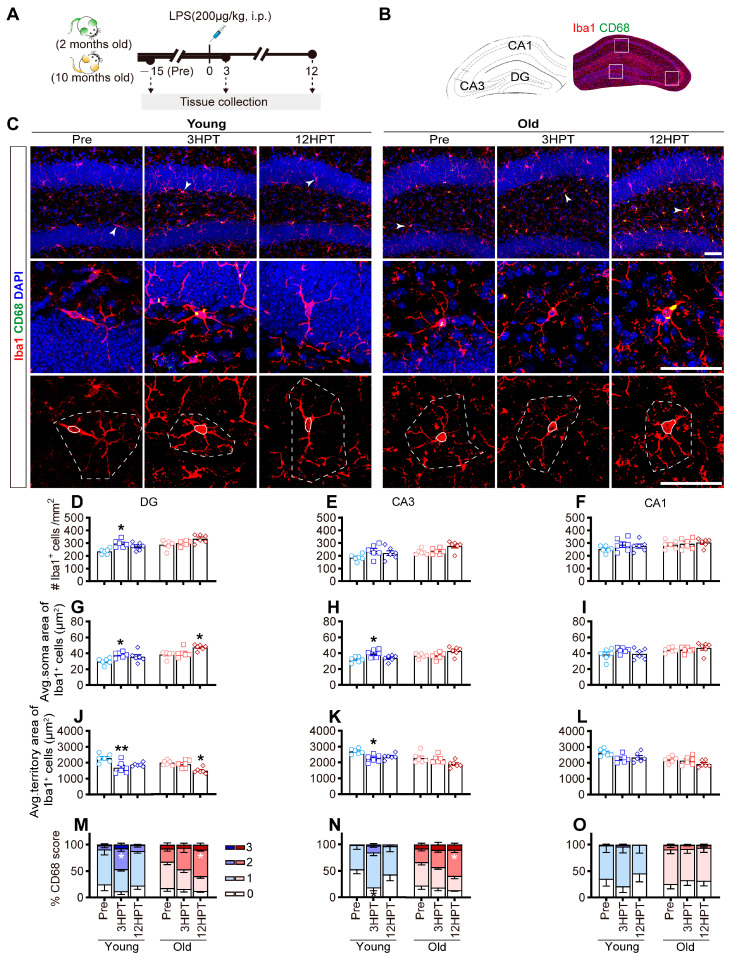
Time-course profiles of phenotypic changes in microglia in the hippocampus of young and old mice. (**A**) Schematic diagram of the treatment schedule and timeline for tissue collection. (**B**) Stereotactic drawings of the hippocampus taken from the Paxinos and Franklin mouse atlas (left) and representative images of immunostaining in coronal sections showing regions of interest (ROIs) corresponding to DG, CA3, and CA1, as indicated by the white box on the right. (**C**) Representative z-projection images of the DG subregion in young and old mice at different time points (pre-treatment and at 3/12 HPT) following staining for Iba1 (red), CD68 (green), and DAPI (blue). Higher magnifications of the selected microglia (arrows) are shown below the corresponding image. Below these images, the split channel for Iba1 is shown, with the soma area and the territory area of microglia delineated by a solid line (soma area) and a dotted line (territory area), respectively. Scale bars = 50 μm. (**D**–**L**) Quantification of microglial density, average microglial soma area, and average microglial territory area in the DG, CA3, and CA1. (**M**–**O**) Quantification of the percentage of CD68^+^ microglia with a score of 0 to 3 in the DG, CA3, and CA1. The data were analyzed using two-way ANOVA (detailed results were listed in Supplementary Table S3) with Tukey’s post hoc test (* *p* < 0.05; ** *p* < 0.01, vs. age-matched baseline (Pre) group). All of the data are expressed as the mean ± standard error of the mean (SEM) (*n* = 6 mice per group). LPS, lipopolysaccharide; HPT, hours post-treatment.

As depicted in Figure 3A–C, three cohorts of mice sacrificed before or at 3/12 HPT were used for the morphological study of hippocampal microglia by immunohistochemistry with the microglial-specific marker Iba1 and the phagocytic marker CD68. At baseline (pre-treatment), microglia in young mice exhibited highly ramified morphology with multiple branches and processes, whereas those in old mice generally covered a smaller area and displayed dystrophic/senescent signs, such as fragmented or beaded processes [39] and increased CD68 expression [40], illustrating age-related phenotypic changes [14]. Following LPS treatment, increases in cell density, soma size, and CD68 expression, as well as the shrink of cellular territory area of microglia, were generally observed in both young and old mice, indicating a microglial response to LPS challenge [10]. However, the temporal pattern of microglial morphological alterations following LPS treatment differed between young and old mice.

At 3 HPT, microglia in young mice exhibited significantly increased cell density (Figure 3D) and soma area (Figure 3G,H) as well as a decreased territory area in the DG/CA3 (Figure 3J,K), whereas these alterations almost vanished at 12 HPT, indicating transient microglial activation following systemic LPS administration. However, in old mice, signs of microglial activation, namely, significantly increased soma size (Figure 3G) and decreased territory area (Figure 3J) in the DG, were observed until 12 HPT, indicating a delayed microglial response to identical LPS stimuli in old mice.

To further characterize the phenotypic profile of microglia, CD68 occupancy within Iba1^+^ cells was assessed on a scale ranging from 0 (indicating low CD68 occupancy) to 3 (indicating high CD68 occupancy), where the higher the given score was, the greater the degree of microglial activation [41]. A transient but significant increase in the percentage of CD68^+^ microglia with a score of 2 in the DG (Figure 3M) and a decrease in the percentage of those with a score of 0 in the CA3 (Figure 3N) were observed in young mice at 3 HPT. Alterations in CD68 occupation were observed in old mice until 12 HPT, as reflected by an increase in the percentage of CD68^+^ microglia with a score of 3 in the DG (Figure 3M) and CA3 (Figure 3N), providing further evidence for the delayed microglial response to the same dose of LPS in old mice.

### 2.4. P16^INK4a^-Positive Senescent Micorglia Accumulate in the Hippocampus with Age but Are Resistant to Modulation

**Figure 4:**
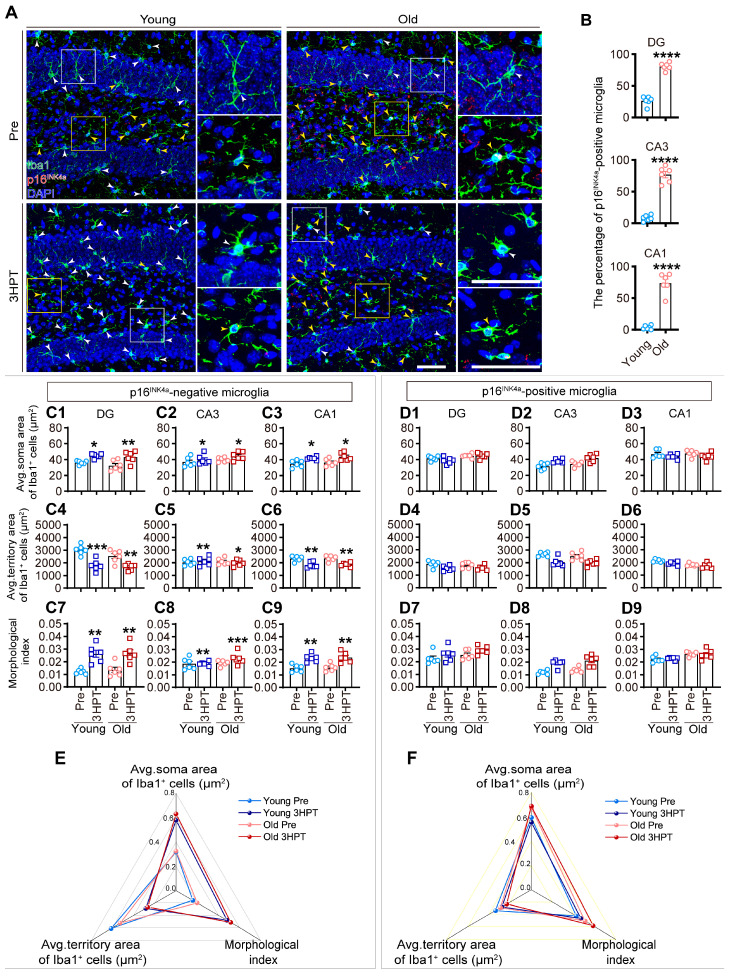
p16^INK4a^-expressing microglia accumulate in the hippocampus of old mice and are resistant to modulation by LPS stimulation. (**A**) Representative images of immunohistochemical staining for Iba1 (green), and p16^INK4a^ (red), and DAPI (blue) in the hippocampus of young and old mice at baseline (pre-treatment) and 3 HPT (hours post-treatment). Higher magnification images of the solid line boxed areas are shown on the right of the corresponding image. The white boxes and arrows indicate p16^INK4a^-negative microglia, whereas yellow boxes and arrows indicate p16^INK4a^-positive microglia. Scale bars = 50 μm. (**B**) Quantitative analysis of the percentage of p16^INK4a^-positive microglia in the hippocampus (DG, CA3, and CA1) of young and old mice at baseline (pre-treatment). The data were analyzed using Student’s two-tailed *t* test (**** *p* < 0.0001, young vs. old). (**C1**–**C9**,**D1**–**D9**) Quantitative analysis of the average microglial soma area, average microglial territory area, and morphological index of p16^INK4a^-negative microglia (**C1**–**C9**) and p16^INK4a^-positive microglia (**D1**–**D9**) in the hippocampus (DG, CA3, and CA1) of young and old mice at baseline (pre-treatment) and 3 HPT. The data were analyzed using two-way ANOVA (detailed results were listed in Supplementary Table S4) with Tukey’s post hoc test (* *p* < 0.05; ** *p* < 0.01; *** *p* < 0.001, vs. the age-matched baseline (Pre) group). All of the data are expressed as the mean ± standard error of the mean (SEM) (*n* = 6 mice per group). (**E**,**F**) Radar charts illustrating the data shown in (**C1**–**C9**,**D1**–**D9**). The data were normalized to 0–1.

To investigate whether microglial senescence is associated with the abnormal microglial response in old mice, we evaluated the morphological alterations of non-senescent and senescent microglia in the hippocampus of young and old mice at baseline (pre-treatment) and 3 HPT by immunostaining for Iba1 and p16^INK4a^ (the cyclin-dependent kinase inhibitor p16, a well-established marker of cellular senescence) (Figure 4A).

We observed that the percentage of p16^INK4a^-positive microglia in the hippocampus of old mice was significantly greater than that in young mice at baseline (pre-treatment) (Figure 4B), indicating that p16^INK4a^-positive microglia predominantly accumulated in the hippocampus of old mice but not in that of young mice.

Interestingly, at 3 HPT, p16^INK4a^-negative microglia in both age groups presented activated phenotypes characterized by a significantly increased soma area (Figure 4C1–C3), decreased territory area (Figure 4C4–C6), and increased morphological index (Figure 4C7–C9), whereas no morphological alterations were observed in p16^INK4a^-positive microglia in either age group (Figure 4D1–D9), suggesting that p16^INK4a^-positive microglia were senescent and resistant to modulation by LPS challenge. Radar charts were constructed to illustrate the changes in the morphological parameters of p16^INK4a^-negative microglia and p16^INK4a^-positive microglia in young and old mice at baseline (pre-treatment) and 3 HPT. Notably, p16^INK4a^-negative microglia at baseline in both age groups presented similar contours characterized by low values of the microglial soma area and morphological index and high values of the territory area but presented contours characterized by high values of the microglial soma area and morphological index and low values of the territory area at 3 HPT (Figure 4E), indicating that p16^INK4a^-negative microglia responded to LPS challenge. However, p16^INK4a^-positive microglia at baseline and 3 HPT displayed similar contours characterized by comparable measurements of microglial parameters (microglial territory area, soma area, and morphological index) in both young and old mice (Figure 4F), indicating that p16^INK4a^-positive microglia are resistant to LPS challenge.

## 3. Discussion

In the present study, we showed that LPS administration (200 μg/kg, i.p.) induced age-dependent delirium-like behavior that was exaggerated in older mice subjected to the same treatment. At 48 HPT, although no delirium-like behavior was observed in any of the LPS-treated mice, aberrant hippocampal neuronal activation associated with prolonged microglial activation was observed in the older mice. Interestingly, in parallel with the dynamics of delirium-like behavior, there was a delayed morphological activation of hippocampal microglia in old mice, which might be associated with the accumulation of senescent microglia that showed minimal morphological activation at 3 HPT. Our findings underscore the importance of optimizing the microglial composition and suggest that senescent microglia targeting is a promising strategy to reduce the vulnerability to inflammatory stimuli.

Inflammation is a well-established trigger of delirium and has been widely recognized as an important integral component of delirium pathophysiology [2]. Here, we demonstrated that LPS administration (200 μg/kg, i.p.) induced attentional and cognitive deficits that are consistent with certain characteristics of delirium observed in humans [42]. Furthermore, only old mice exhibited attentional and cognitive impairments at 6 HPT, indicating age-related differences in delirium-like behavior. Thus, the mouse model utilized in our present study is suitable for elucidating the mechanisms underlying the age-related differences in inflammation-induced delirium. Histological assessments revealed that although no delirium-like behavior was observed in any of the LPS-treated mice at 48 HPT, aberrant hippocampal neuronal activation, as evidenced by reduced c-Fos^+^ neurons, was observed in the older mice. Previous studies have shown that the abnormalities in neuronal activity are likely the key mechanisms mediating the occurrence of delirium-like behavior [36,37] and diverse neurological deficits [43]. Thus, we speculate that the insidious abnormality of neuronal activity in the cognitive brain region following delirium-like behavior may provide valuable clues for identifying underlying reasons for the increased vulnerability to future neurocognitive impairments in older individuals.

In line with previous studies showing the central role of microglia in the development of delirium [18,19] and efforts to investigate microglial dynamics in age-related neurobehavioral and neuroinflammatory diseases [21,44], we observed that the behavioral abnormalities following LPS administration were accompanied by alterations in the microglial phenotype. Interestingly, the temporal profiles of the phenotypic changes in microglia differed between young and old mice. Specifically, microglia in young mice displayed transient activation at 3 HPT, whereas microglia in old animals did not present overt morphological changes at this time point but showed pronounced activation at 12 HPT. Adequate and appropriate microglial activation often facilitates injury repair by clearing invading pathogens and cell debris [45], whereas inappropriate (untimely/persistent) microglial activation may lead to insufficient/excessive phagocytosis and hasten disease progression [9]. Thus, we propose that this delayed response of hippocampal microglia in old mice is unlikely to be beneficial to the aged brain and might be associated with the exacerbated delirium-like behavior observed in old mice. Additionally, microglia in young animals had almost returned to baseline levels at 48 HPT, whereas those in old mice exhibited prolonged activation, suggesting that aging affects not only the initial responsiveness of microglia but also their capacity to fully recover, at least at the time points and ages assessed in our present study. Given the critical role of aberrant microglial activation in neuronal injury (including changes in neuronal activity) [43] and the observed link between such anomalies in the hippocampus of old LPS-treated mice, we speculate that prolonged microglial activation in old mice might lead to long-term neurological impairments.

Microglial senescence refers to the state of cellular aging in microglia and is characterized by irreversible cell cycle arrest mediated by cyclin-dependent kinase inhibitors (including p16^INK4a^), along with other cellular/molecular changes that render them dysfunctional [20,46]. The morphological transformation of senescent microglia is termed microglial dystrophy, which is characterized by de-ramification and fragmentation of their processes, as well as abnormal phagocytic function [46]. Using single-cell RNA sequencing (scRNA-seq), Hammond et al. revealed the remarkable diversity and heterogeneity of microglia, demonstrating that while chronological age is undoubtedly the natural driver of microglial senescence, it is not indispensable [47]. Similarly, using scRNA-seq, Talma et al. identified two distinct p16-expressing microglial subpopulations, with one accumulating with age and the other already present in young individuals [48]. In agreement with these studies, we observed the coexistence of ramified microglia and dystrophic microglia in the hippocampus of both young and old mice. These results, along with the result of coimmunostaining for Iba1 and p16^INK4a^, which revealed the coexistence of p16^INK4a^-positive microglia and p16^INK4a^-negative microglia, provide histological evidence and suggest that microglial senescence is a phenomenon that is not exclusively confined to older animals, although the aging process contributes to changes in the microglial phonotype and the accumulation of microglia expressing p16^INK4a^. The accumulation of senescent microglia, in conjunction with their compromised immune functions and perturbed interactions with other brain cells, has previously been shown to potentially play a critical role in the etiology of neurodegenerative diseases [20,49,50,51,52]. However, whether the delayed response of hippocampal microglia in old mice is underpinned by microglial senescence remains enigmatic. Interestingly, our results demonstrated that at 3 HPT, non-senescent microglia in both age groups displayed approximate activation, characterized by a significantly increased soma area, decreased territory area, and increased morphological index; moreover, no significant morphological alterations indicative of activation were observed in the senescent microglia. These results suggest that the increase in the absolute number of senescent microglia might be a key substrate for the abnormal microglial response in elderly animals. In light of recent reports showing that eliminating or rejuvenating senescent microglia may represent alternative treatment strategies for neurodegenerative diseases [49,50,53], it is reasonable to speculate that interventions intended to halt or delay microglial senescence (such as the transgenic *p16-InkAttac* mice and senolytic drugs) might lead to actual reductions in the incidence of delirium (not just in elderly individuals) as well as delirium-related neurocognitive sequelae.

To our knowledge, this is the first report suggesting that the age-related risk for LPS-induced delirium-like behavior accompanied by different dynamics of the microglial response might implicate a distinct composition of microglia in the hippocampus. Our speculation is particularly supported by a recent study showing that systemic targeting of p16^INK4a^-positive senescent cells may remodel the age-affected brain immune cell composition (a significant reduction in the abundance of nonactivated or ‘homeostatic microglia’, which coincided with the expansion of activated resident microglia and infiltrating inflammatory cell populations) to a more youthful state while improving cognitive function [54]. Given the limited efficacy of pharmacological treatments for delirium and its high prevalence during acute illness, the integration of our observations with the above findings provides valuable insight into new strategies to reduce age-dependent vulnerability to inflammatory challenges. In addition, the nutritional status in rodent models is significant, in particular, when investigating effects on lifespan and neurological conditions [55]. We previously demonstrated that excessive high-fat diet consumption induced neurobehavioral abnormalities, neuronal structural alterations, and microglial overactivation in mice [9,10], suggesting a detrimental effect of unhealthy dietary patterns on neurological health. Conversely, beneficial effects of certain dietary interventions on neurological health have also been observed in several independent studies. For example, a ketogenic diet was a prime therapy to control epilepsia until the predominance of pharmacological interventions arose [56]. Coconut oil has the potential to act as an alternative energy source for the brain, potentially enhancing cognitive function in neurodegenerative conditions such as Alzheimer’s disease [57]. Essential micronutrients such as lithium have also garnered significant attention for their therapeutic potential in neurological disorders [58]. As the global population ages, it is becoming more crucial to incorporate dietary modifications into a comprehensive strategy for managing and preventing neurological disorders, like delirium. Furthermore, our current study demonstrated that LPS induced delirium-like behavior in both young and elderly individuals, underscoring the role of toxins in the development of neurological disorders. Beyond endotoxins, environmental pollutants, like aluminum and heavy metals, have also been implicated in the pathogenesis of such disorders [59,60]. Thus, implementing appropriate detoxification strategies and minimizing toxin exposure are reasonable measures for preventing neurological disorders, especially in industrialized and urban areas.

Although our research has important implications, several limitations of our study cannot be ignored. First, the present study was limited to males to avoid the potential confounding effects of estrous cycle variability and hormonal fluctuations in females [61], and further research is warranted to investigate the potential sex differences in delirium occurrence and microglial responses. Second, our work revealed only parallel observations of behavioral performance and histological changes, and pharmacological strategies (such as the colony-stimulating factor 1 receptor inhibitor PLX5622) could be used to regenerate microglia in old mice to investigate the exact upstream and downstream relationships. Third, delirium results from pathological changes, affecting, in particular, the hippocampus, frontal cortex, amygdala, and brainstem [62], and further work should explore the heterogeneity in microglial response across different brain regions to determine the specific contribution of each implicated brain area to the pathogenesis of delirium.

## 4. Materials and Methods

### 4.1. Animals and Experimental Design

Male C57BL/6 mice of 2 months and 10 months of age (42 in total for each age; for simplicity, referred to as Young and Old) were purchased from Hangzhou Zi yuan Experimental Animal Technology Co., Ltd. (Hangzhou, China; SCXK (ZHE) 2019-0004). These animals were maintained in a specific pathogen-free environment, with three to four age-matched mice housed per cage under standard conditions (7 a.m. to 7 p.m. light cycle, 22 °C, 55% humidity), and provided with ad libitum access to food (carbohydrate: 61.1%, protein: 23.4%, fat: 15.5%; GB 14924.3-2010 [63]) and water.

After a one-week period of acclimatization, 24 young mice and 24 old mice were randomly selected and assigned to the NS or LPS group (termed the Y-NS, Y-LPS, O-NS, or O-LPS group; *n* = 12 mice per group). To avoid stress arising from manipulation and novelty during testing, the mice were handled by the same experimenter for 5 consecutive days for 5 min each time before beginning the experiments. As illustrated in Figure 1A, these 48 mice were transferred to polycarbonate test cages (dimensions: 26 cm × 15.5 cm × 12.5 cm) on the first experimental day and were individually housed until the end of the study. A high-resolution video camera (Luowice, Guangzhou, China) was placed on one sidewall of each cage to record the mouse’s behavior. Following 1 h of acclimation, the mice were subjected to baseline behavioral tests, which began at approximately 6 p.m. (designated Pre) and were completed within 1 h. On the second experimental day, the mice were treated by i.p. injection of NS or LPS (200 μg/kg) at approximately 9:00 a.m. followed by behavioral tests repeated at 3, 6, 12, 24, and 48 HPT. At 1.5 h after the last behavioral test, the mice were sacrificed, and their brains were collected for the study of c-Fos expression [64].

As illustrated in Figure 3A, a separate batch of mice (18 mice of each age, 36 in total) were randomly divided into three cohorts and sacrificed before (pre-treatment) or at 3/12 h post i.p. injection of LPS (200 μg/kg) for brain collection and immunohistochemistry studies (*n* = 6 mice per time point for each age).

### 4.2. LPS/NS Treatment

The mice in the LPS group were treated with a single i.p. injection of 200 μg/kg LPS (*Escherichia coli* O55:B5; Sigma, St. Louis, MO, USA; #L2880) dissolved in physiological saline (sterile 0.9% NaCl) [30,65]. The mice in the NS group received an equal volume of saline via intraperitoneal injection.

### 4.3. Evaluation of Sickness Score

The sickness behavior of each mouse was monitored before and at 3, 6, 12, 24, and 48 HPT by two trained researchers blinded to the experimental groups using an established 12-point scale of sickness behavior [66,67]. On this scale, each animal was assessed for response to finger pokes (4—normal; 3—slightly decreased; 2—severely decreased; 1—minimal response; 0—no response/dead), signs of encephalopathy (4—normal gait; 3—tremors or staggering; 2—twisting movements; 1—falling or turning), and general appearance (4—normal, with a reduction of 1 for each observed occurrence of piloerection, periorbital exudates, respiratory distress, or diarrhea). A score of 12 indicates a healthy mouse with normal activity, whereas a score of 0 reflects a moribund condition.

### 4.4. Behavioral Tests

Both the BFT and NORT were deployed pre-treatment and at 3, 6, 12, 24, and 48 HPT for the evaluation of attention and cognition.

#### 4.4.1. BFT

The BFT was used to assess a mouse’s ability to find a buried cereal reward, a behavior that relies on intact attention and organized thinking [33]. This test was carried out as previously described with some modifications [31,32]. Specifically, three days prior to LPS treatment, each mouse was given a sweetened cereal pellet to familiarize themselves with the odor of the food. In the BFT, the mouse was introduced into the center of the test cage (26 cm × 15.5 cm × 12.5 cm) with 3 cm of clean bedding, where a sweetened cereal pellet had been randomly buried 0.5 cm below the surface of the bedding. The latency to the pellet was defined as the time from placing the mouse in the cage until the mouse found and grasped the pellet in the forepaws and/or teeth [31]. Once the pellet was found by the mouse, it was removed immediately to prevent any potential impact on the mouse’s subsequent behavior resulting from pellet consumption. If the mouse failed to find the pellet within 5 min, the latency was recorded as 300 s for that mouse. Video analysis was performed by four trained assessors blinded to the experimental groups, and the average was calculated to represent each mouse’s data.

#### 4.4.2. NORT

The NORT, which exploits rodents’ natural tendency to explore novel stimuli, was used to assess working memory, a component of executive cognitive function [30]. The procedure was conducted as previously described [35] with slight modifications. As illustrated in Figure 1A, in the test performed pre-treatment, two identical objects (cubes made of semitransparent solid rubber; 4 cm × 4 cm × 4 cm) were fixed symmetrically on the left and right sides of the test cage (26 cm × 15.5 cm × 12.5 cm), and the mouse was allowed to explore freely for 10 min. In the test performed at 3 HPT, the object on the left was substituted with a novel object, and the right one remained and served as a familiar object in this phase. In the tests performed at 6, 12, 24, and 48 HPT, the familiar object in the previous study was replaced with a novel object, and the other object remained to serve as the familiar object in the corresponding phase. The objects presented in the test after LPS treatment varied in shape and texture and were salient from the background, as listed in Table 1. Object exploration was defined as the mouse sniffing the object or touching the object while looking at it, with the distance between the nose and the object within 2 cm; climbing onto the object (unless the mouse sniffed the object it climbed on) or chewing the object was not considered as exploratory behavior [68]. The time spent exploring the old/novel object for 10 min was determined by four experimenters who were blind to the experimental conditions. The novel object preference was reported as a percentage and calculated as [(time spent with novel object)/(time spent with novel object and original object) × 100%]. A decrease in novel object preference indicated working memory impairment.

### 4.5. Tissue Collection

At each designated time point, the mice were deeply anesthetized with sodium pentobarbital (100 mg/kg, i.p.) and transcardially perfused with 20 mL of pre-cooled 0.1 M phosphate-buffered saline (PBS) to wash out the blood, followed by 40 mL of 4% paraformaldehyde (PFA) in 0.1 M PBS to fix the brain tissue. The brains were isolated and postfixed in 4% PFA at 4 °C overnight before being cryoprotected in 30% sucrose. Upon sinking, the brains were embedded in optimal cutting temperature (OCT) compound and stored at −80 °C until further use. Serial coronal sections (40 μm thick) were obtained with a freezing microtome (CM1900, Leica Biosystems, Richmond, IL, USA) and stored in cryoprotectant solution (30% ethylene glycol, 25% glycerin in 0.1 M phosphate buffer) at −20 °C until staining.

### 4.6. Immunohistochemistry

Two to three brain sections containing the hippocampus (320 μm intervals, bregma −1.46 to −2.46 mm) were selected from each animal by referencing the Allen Mouse Brain Atlas [69]. Free-floating sections were permeabilized in PBS containing 0.1% Triton X-100 for 30 min, blocked with blocking solution for 2 h, and incubated with primary antibodies diluted in blocking serum overnight at 4 °C. The sections were then incubated with secondary antibodies for 2 h at room temperature. The following antibodies were used for immunohistochemistry: rabbit anti-ionized calcium-binding adaptor molecule 1 (Iba1, for microglia; 1:1000; 019-19741; Wako, Osaka, Japan), goat anti-Iba1 (for microglia; 1:600; 011-27991; Wako), rat anti-cluster of differentiation 68 (CD68, for activated microglia; 1:1500; MCA1957; Bio-Rad, Oxford, UK), rabbit anti-CDKN2A/p16INK4a (p16^INK4a^, for senescent cells; 1:500; ab211542; Abcam, Waltham, MA, USA), mouse anti-NeuN (for neurons; 1:250; MAB377; Millipore, Burlington, MA, USA), and rabbit anti-c-Fos (for activated neurons; 1:200; cat#2250; Cell Signaling Technology, Danvers, MA, USA). Alexa Fluor 488-conjugated donkey anti-goat IgG (1:1000, ab150129, Abcam), Alexa Fluor 594-conjugated donkey anti-rat IgG (1:1000, ab150156, Abcam), Alexa Fluor 568-conjugated donkey anti-mouse IgG (1:1000, A-10037, Invitrogen, Waltham, MA, USA), and Alexa Fluor 647-conjugated donkey anti-rabbit IgG (1:1000, A-31573, Thermo Fisher Scientific, Waltham, MA, USA) were used as secondary antibodies. The cell nuclei were stained in the dark with 4,6-diamidino-2-phenylindole (DAPI, 1:600; C1027; Beyotime, Shanghai, China). Negative controls were treated using the same protocol without primary antibodies to assess nonspecific labeling.

### 4.7. Quantitative Immunohistochemistry Image Analysis

Images were obtained using a confocal microscope (FV3000, Olympus, Tokyo, Japan) with 20×, 40×, or 60× objectives and a digital slide scanner (Olympus VS200, Japan) with a 20× objective. The ROIs (DG, CA3, and CA1 regions of the hippocampus) were delineated according to the cell densities identified by DAPI and referenced to the Allen Mouse Brain Atlas [69]. All of the confocal stacks were acquired with a resolution of 1024 × 1024 pixels and a z-step of 1 µm to generate confocal stacks of ~20 μm per image. All acquisition and display settings were identical between the groups, and the settings were optimized using the O-LPS group. ImageJ software 1.52a (US National Institutes of Health, Bethesda, MD, USA) was used for image analysis by an observer blinded to the experimental design.

For the quantitative assessment of microglia, maximum intensity projections were generated, and only Iba1^+^ cells with a DAPI^+^ nucleus were analyzed (*n* = 6 animals/ group; *n* = 2~3 images/animal). As described in previous reports [9,10,11,14,41,70,71,72], the following parameters were analyzed: the density of Iba1^+^ cells (defined as the number of Iba1^+^ cells per area); the average soma area of Iba1^+^ cells; the average territory area of Iba1^+^ cells (defined as the area delineated by the outermost points of the dendritic processes of an Iba1^+^ cell); and the percentage of CD68^+^ microglia with scores of 0–3 (defined as the proportion of CD68^+^ Iba1^+^ cells with scores of 0–3 among all Iba1^+^ cells). A score of 0 indicated scarce CD68 staining; a score of 1 indicated punctate CD68-positive signals; a score of 2 indicated CD68 staining covering 1/3 to 2/3 of the total area; and a score of 3 indicated a CD68-stained area greater than 2/3 of the total area. The morphological index was calculated by dividing the average soma area by the average territory area for each image [73].

For the quantitative assessment of neuronal activation, slices were stained for Iba1, c-Fos, and NeuN, and z-stacks were taken in the hippocampus (*n* = 6 animals/group; *n* = 2~3 images/animal). The numbers of c-Fos^+^NeuN^+^ cells and NeuN^+^ cells within the ROIs were manually counted in 20 μm z-stacked confocal images using ImageJ. The results are presented as the percentage of c-Fos^+^NeuN^+^ cells among all NeuN^+^ cells.

For the quantitative evaluation of the percentage of p16^INK4a^-positive microglia (defined as the proportion of p16^INK4a+^Iba1^+^ cells among all Iba1^+^ cells), slices were stained for Iba1 and p16^INK4a^, and z-stacks were taken from the hippocampus (*n* = 6 animals/group; *n* = 2~3 images/animal). The numbers of p16^INK4a+^Iba1^+^ cells and Iba1^+^ cells within the ROIs were manually counted in 40 μm z-stacked confocal images using ImageJ.

### 4.8. Statistical Analysis

GraphPad Prism 9.4.0 (GraphPad Software Inc., San Diego, CA, USA) and Origin software (Version 2022, Origin Lab Corp., Northampton, MA, USA) were used for statistical analysis and plotting. All of the data are expressed as the mean ± standard error of the mean (SEM), and n represents the number of animals. The level of statistical significance between groups was determined by two-way analysis of variance (ANOVA) followed by a post hoc test (Tukey’s or Sidak’s test), two-tailed Student’s *t* test, or one sample *t* test, as appropriate. *p* < 0.05 was considered to indicate statistical significance. Correlations between parameters were evaluated by Pearson’s correlation test.

## Figures and Tables

**Table 1:** Novel objects employed in the NORT.

Detection Time Points	Novel Object Description	Novel Object Size
3 HPT	A tower made of plastic red and blue Lego bricks	6 cm high, 3 cm deep, and 3 cm wide
6 HPT	A transparent glass bottle filled with green pieces of paper scraps	6 cm high, 2.5 cm diameter
12 HPT	A cylinder made of semi-transparent solid rubber with a red core	4.5 cm high, 4 cm diameter
24 HPT	A tower made of plastic blue and white Lego bricks	7 cm high, 3 cm deep, and 3 cm wide
48 HPT	A plastic bottle containing water with red and green paper wrapped around the outer surface	4.5 cm high, 3.5 cm diameter

## Data Availability

Data contained within the paper are available from the authors upon reasonable request.

## Supplementary Materials

The supporting information can be downloaded at: https://www.mdpi.com/article/10.3390/ijms26052055/s1.
