# Supplementary material for: Age-Related Differences in Lipopolysaccharide-Induced Delirium-like Behavior Implicate the Distinct Microglial Composition in the Hippocampus
Source: Int J Mol Sci. 2025 Feb 26;26(5):2055. doi: 10.3390/ijms26052055 (PMC11900323; doi:10.3390/ijms26052055)
Supplement: Supplementary file 1 [file ijms-26-02055-s001.zip › ijms-3406333-supplementary.pdf]

Table S1. Two-way repeated-measures ANOVA table with time and group/treatment as the main factors.

	time			*group*			*time × group*		
	F	***df***	***p***	***F***	***df***	***p***	***F***	***df***	***p***
Sickness score	52.74	1.988,87.49	< 0.0001	21.75	3,44	< 0.0001	16.78	15,220	< 0.0001
	time			***treatment***			***time × treatment***		
	F	***df***	***p***	***F***	***df***	***p***	***F***	***df***	***p***
Young									
Latency to pellet in the BFT	10.68	1.956,43.03	0.0002	14.68	1,22	0.0009	9.301	5,110	< 0.0001
Novel object preference (%) in the NORT	17.77	4,88	< 0.0001	0.04810	1,22	0.08284	2.981	4,88	0.0233
Old									
Latency to pellet in the BFT	27.29	2.138,47.03	< 0.0001	33.13	1,22	< 0.0001	22.19	5,110	< 0.0001
Novel object preference (%) in the NORT	11.29	2.247,49.43	< 0.0001	39.66	1,22	< 0.0001	5.454	4,88	0.0006

Table S2. Two-way ANOVA table with age and treatment as the main factors.

	age			*treatment*			*age × treatment*		
	F	***df***	***p***	***F***	***df***	***p***	***F***	***df***	***p***
DG									
% c-Fos+ neurons	12.24	1,20	0.0023	15.86	1,20	0.0007	5.633	1,20	0.0278
Microglial density	18.29	1,20	0.0004	4.283	1,20	0.0517	0.6403	1,20	0.4330
Avg. soma area	10.42	1,20	0.0042	9.889	1,20	0.0051	9.328	1,20	0.0063
Avg. territory area	42.97	1,20	<0.0001	17.74	1,20	0.0004	4.319	1,20	0.0508
CA3									
% c-Fos+ neurons	11.98	1,20	0.0025	12.73	1,20	0.0019	7.662	1,20	0.0119
Microglial density	12.44	1,20	0.0021	5.623	1,20	0.0279	0.7248	1,20	0.4047
Avg. soma area	6.246	1,20	0.0213	8.566	1,20	0.0083	3.024	1,20	0.0974
Avg. territory area	31.01	1,20	<0.0001	9.525	1,20	0.0058	3.301	1,20	0.0843
CA1									
% c-Fos+ neurons	7.359	1,20	0.0134	13.88	1,20	0.0013	7.046	1,20	0.0152
Microglial density	1.139	1,20	0.2985	0.2729	1,20	0.6071	0.0005	1,20	0.9821
Avg. soma area	0.1689	1,20	0.6854	17.43	1,20	0.0005	0.3328	1,20	0.5704
Avg. territory area	18.20	1,20	0.0004	9.792	1,20	0.0053	2.950	1,20	0.1013

Table S3. Two-way ANOVA table with age/score and time as the main factors.

	age			*time*			*age × time*		
	F	***df***	***p***	***F***	***df***	***p***	***F***	***df***	***p***
DG									
Microglial density	16.87	1,30	0.0003	7.590	2,30	0.0022	3.513	2,30	0.0426
Avg. soma area	22.62	1,30	< 0.0001	7.668	2,30	0.0020	3.459	2,30	0.0445
Avg. territory area	4.064	1,30	0.0528	11.84	2,30	0.0002	5.564	2,30	0.0088
CA3									
Microglial density	7.228	1,30	0.0116	4.292	2,30	0.0229	2.141	2,30	0.1352
Avg. soma area	6.506	1,30	0.0161	3.496	2,30	0.0432	5.740	2,30	0.0077
Avg. territory area	16.28	1,30	0.0003	7.384	2,30	0.0025	3.459	2,30	0.0642
CA1									
Microglial density	3.356	1,30	0.0769	1.178	2,30	0.3217	0.4206	2,30	0.6605
Avg. soma area	6.182	1,30	0.0187	0.6834	2,30	0.5162	1.335	2,30	0.2785
Avg. territory area	15.44	1,30	0.0005	4.809	2,30	0.0154	1.656	2,30	0.2079

	score			*time*			*score × time*		
	F	***df***	***p***	***F***	***df***	***p***	***F***	***df***	***p***
Young									
DG	42.73	3,60	< 0.0001	7.840e-010	2,60	> 0.9999	4.843	6,60	0.0004
CA3	50.20	3,60	< 0.0001	2.548e-009	2,60	> 0.9999	3.684	6,60	0.0035
CA1	28.23	3,60	< 0.0001	1.772e-009	2,60	> 0.9999	0.8837	6,60	0.5125
Old									
DG	30.28	3,60	< 0.0001	2.762e-010	2,60	> 0.9999	3.865	6,60	0.0025
CA3	25.63	3,60	< 0.0001	3.193e-008	2,60	> 0.9999	3.858	6,60	0.0025
CA1	53.97	3,60	< 0.0001	6.450e-010	2,60	> 0.9999	0.2251	6,60	0.9671

Table S4. Two-way ANOVA table with age and time as the main factors.

	age			*time*			*age × time*		
	F	***df***	***p***	***F***	***df***	***p***	***F***	***df***	***p***
p16^{INK4a}-negative microglia									
DG									
Avg. soma area	1.514	1,20	0.2328	21.14	1,20	0.0002	0.3287	1,20	0.5728
Avg. territory area	3.666	1,20	0.0700	41.53	1,20	< 0.0001	1.251	1,20	0.2767
Morphological index	0.1533	1,20	0.6996	35.80	1,20	< 0.0001	0.04598	1,20	0.8324
CA3									
Avg. soma area	4.249	1,20	0.0525	20.68	1,20	0.0020	0.07972	1,20	0.7806
Avg. territory area	0.6490	1,20	0.4300	26.72	1,20	< 0.0001	0.1786	1,20	0.6771
Morphological index	3.067	1,20	0.0952	40.47	1,20	< 0.0001	0.02053	1,20	0.8875
CA1									
Avg. soma area	1.285	1,20	0.2705	18.21	1,20	0.0004	0.01269	1,20	0.9114
Avg. territory area	0.08786	1,20	0.7700	35.06	1,20	< 0.0001	0.02028	1,20	0.8882
Morphological index	0.2177	1,20	0.6458	35.93	1,20	< 0.0001	0.0002	1,20	0.9880
p16^{INK4a}-positive microglia									
DG									
Avg. soma area	12.08	1,20	0.0024	0.9751	1,20	0.3352	1.358	1,20	0.2576
Avg. territory area	0.09253	1,20	0.7641	10.29	1,20	0.0044	0.4863	1,20	0.4936
Morphological index	2.824	1,20	0.1084	3.935	1,20	0.0612	0.01157	1,20	0.9154
CA3									
Avg. soma area	3.348	1,20	0.0822	3.185	1,20	0.0895	0.2193	1,20	0.6447
Avg. territory area	0.1678	1,20	0.6864	0.0034	1,20	0.9543	0.9387	1,20	0.3442
Morphological index	2.809	1,20	0.1093	1.794	1,20	0.1955	1.545	1,20	0.2283
CA1									
Avg. soma area	0.07212	1,20	0.7910	3.907	1,20	0.0620	0.03115	1,20	0.8617
Avg. territory area	19.12	1,20	0.0003	5.404	1,20	0.0307	0.7084	1,20	0.4099
Morphological index	12.89	1,20	0.0018	0.0061	1,20	0.9386	0.2734	1,20	0.6068
